# Testicular neoplasms: the interrelationships of serum levels of microRNA-371a-3p (M371) and classical tumor markers with histology, clinical staging, and age—a statistical analysis

**DOI:** 10.1007/s00432-023-04664-8

**Published:** 2023-03-04

**Authors:** Klaus-Peter Dieckmann, Cansu Dumlupinar, Francesca Grobelny, Julia Utschig, Markus Klemke, El Moeiz Ahmed Saad, Christian Wülfing, Uwe Pichlmeier, Hendrik Isbarn, Gazanfer Belge

**Affiliations:** 1 Department of Urology, Asklepios Klinik Altona, 22763 Hamburg, Germany; 2 Department of Tumour Genetics, Faculty of Biology and Chemistry, University of Bremen, Leobener Strasse 2/FVG, 28359 Bremen, Germany; 3 Faculty of Medicine, University of Hamburg, Hamburg, Germany; 4 Medilys Labor, Asklepios Klinik Altona, 22763 Hamburg, Germany; 5 Institute of Medical Biometry and Epidemiology, Universitätsklinikum Eppendorf, 20251 Hamburg, Germany; 6 Martini Klinik, Universitätsklinikum Eppendorf, 20251 Hamburg, Germany

**Keywords:** Testicular neoplasms, Seminoma, Nonseminoma, MicroRNA-371a-3p, Clinical stage, Alpha fetoprotein, Beta HCG, Lactate dehydrogenase

## Abstract

**Purpose:**

In testicular neoplasms, the interrelationship of elevations of the novel serum tumor marker microRNA-371a-3p (M371) and traditional markers with other clinical features is still incompletely understood. The present study evaluated marker expression rates in relation to various other clinical parameters.

**Methods:**

The following data were retrospectively registered from 641 consecutive patients with testicular neoplasms: histology, such as seminoma (*n* = 365), nonseminoma (*n = *179), benign tumor (*n = *79), other malignant tumor (*n = *18); patients age (years); clinical stage (CS1, CS2a/b, CS2c, CS3); and preoperative elevation of beta HCG, AFP, LDH, M371 (yes/no). Descriptive statistical methods were employed with comparisons of various subgroups to disclose associations of marker expression rates with age, histology and CS, and of age with histology.

**Results:**

The histologic subgroups revealed significantly different expression rates of tumor markers. M371 performed best with expression rates of 82.69% and 93.58% in seminoma and in nonseminoma, respectively. In germ cell tumors, all markers had significantly higher expression rates in metastasized stages than in localized disease. All markers except LDH have significantly higher expression rates in younger than in older patients. Nonseminoma is most prevalent in the youngest age category, seminoma predominates in patients > 40 years, other malignancies were restricted to patients > 50 years.

**Conclusion:**

The study documented significant associations of serum marker expression rates with histology, age and clinical staging, with highest rates in nonseminomas, young age and advanced clinical stages. M371 showed significantly higher expression rates than other markers suggesting its superior clinical usefulness.

## Introduction

The factors histology, clinical stage (CS), serum tumor marker elevation, and patient age represent the traditional mainstay of clinical decision-making in testicular neoplasms according to recent guide-lines (Kliesch et al. 2021; Oldenburg et al. 2022; Gilligan et al. 2019). In contrast to other malignancies, particularly hematological neoplasms (Khoury et al. 2022), molecular genetic features have only gained marginal clinical importance in testicular tumors to date. Searching for isochromosome 12p for identification of germ cell tumors (GCTs) is the only molecular genetic tool employed in selected cases of testis tumors (Wyvekens et al. 2022). Histology forms the basis for the first strategic decision in the management of testicular neoplasms, because benign tumors do not need further treatment after surgery while the management of GCTs needs to be tailored to seminomatous or nonseminomatous histology and secondly to clinical stage (Cheng et al. 2018; Chovanec and Cheng 2022). Elevation of traditional serum tumor markers alpha fetoprotein (AFP), beta human chorionic gonadotropin (bHCG) and lactate dehydrogenase (LDH) is a typical feature of nonseminomatous GCTs, while it is less frequently found in seminomas and it is not observed in other testicular neoplasms (Leão et al. 2018). The extent of serum tumor marker elevation is paramount for prognostic grouping according to the International Germ Cell Cancer Consensus Group (IGCCCG) (Gillessen et al. 2021; Beyer et al. 2021). Recently, the novel serum tumor marker microRNA-371a-3p (M371) has been reported to outperform the traditional markers (Dieckmann et al. 2019a; Fankhauser et al. 2022). However, despite continuously increasing numbers of scientific reports (Leão et al. 2021; Almstrup et al. 2020; Regouc et al. 2020; Konneh et al. 2022), the full spectrum of features of this new marker is still little understood. Finally, patient age is a critical factor for clinical decision-making, because in the elderly, toxicity of treatment is much greater and cure rates are clearly inferior (Terbuch et al. 2019; Miller et al. 2017; Gillessen et al. 2021).

Most likely, the factors serum tumour marker expression, histology, clinical staging, and age represent a biological network with manifold interrelationships between each other. However, the interplay of these factors is only scantily explored (Dieckmann et al. 2019b). Accordingly, we evaluated four particular clinical scenarios, all of which represent associations of clinical factors that are frequently encountered in clinical practice, but the formal evidence derived from contemporary case series is still limited: *(#1)* Patient age has a bearing on histology of the primary, with quite divergent age predispositions of the various histologic subtypes of testicular neoplasms. *(#2)* Histology of testicular neoplasms impacts the frequencies of elevations of serum tumor markers with high expression rates in nonseminomas, moderate rates in seminoma and no expression in non-germ cell tumors. M371 is expected to be expressed in both subgroups of GCTs but not in non-germ cell tumors. (*#3)* Frequencies of tumor marker expression in GCTs are associated with clinical stages with higher frequencies of elevations in advanced clinical stages. *(#4)* Frequencies of marker elevations are inversely associated with age.

To test these clinical associations, we systematically analyzed the four parameters in a large series of consecutive patients with testicular tumors.

## Materials and methods

### Patients recruitment, data procurement

The patient population of this retrospective study consisted of all male subjects aged 17–98 years, diagnosed with testicular new growths while undergoing surgery in two Hamburg based urologic departments (Albertinen-Krankenhaus and Asklepios Klinik Altona) during 2012–2021. Patients with previous chemotherapy were excluded. The following data were secured from hospital-based electronic case files: patient’s age (years); histology of the surgical specimen categorized as seminoma (SE), nonseminoma (NS), benign tumor (BT), malignant tumor other than GCT (OM); clinical stage (CS) at diagnosis (only in GCTs); and preoperative elevation of serum tumor markers bHCG, AFP, LDH, M371. The majority of the patients had been included in previous reports on other issues (Dieckmann et al. 2019a, 2019b, 2018, 2022a).

Histological diagnoses were derived from electronic documents without central pathology review. Clinical stages were filed as CS1; CS2a/b; CS2c; and CS3 according to modern guide-lines (Kliesch et al. 2021). Serum tumor markers bHCG, AFP, and LDH were measured in hospital laboratories according to institutional standard operating procedures. As test kits were repeatedly replaced for economic reasons during 2012–2021, the normal limits of tumor marker levels had to be adjusted, accordingly. Therefore, we restricted the study of serum tumor markers to a dichotomized analysis (i.e., elevation above the upper limit of norm [ULN] yes/no). The novel marker M371 was measured by quantitative polymerase chain reaction with quantification in relation to endogenous miR-30b-5p, as detailed earlier (Dieckmann et al. 2019a). M371 measurement results were originally documented as relative quantity (RQ) values defining RQ = 5 as ULN. For reasons of methodological conformity, only dichotomized results were recorded (elevated yes/no).

The Ethikkommission der Ärztekammer Hamburg gave ethical approval (PV7288). The need for informed consent of patients was waived, because merely anonymized data were subject to the investigation. All study activities fully complied with the Declaration of Helsinki of the World Medical Association as amended by the 64th General Assembly, October 2013.

### Statistical analysis

All case-related data were originally filed in a commercially available data base (MS Excel, version 2017) after thorough data validation. Final statistical analysis was conducted with SAS software package version 9.4 (SAS Institute, Cary, NC, USA) on windows platform.

Descriptive statistical analysis of nominal variables was accomplished by calculating absolute frequencies, percentages and 95% exact Clopper–Pearson confidence intervals (CIs).

Descriptive statistical analysis of continuous variables involved calculation of median, first quartile (Q1), third quartile (Q3), interquartile range (IQR), minimum, and maximum. Chi-square tests were used to compare contingency tables of nominal variables. Cochran–Armitage trend tests were applied to assess whether marker expression rates increase with clinical stages or with increasing age categories. The Kruskal–Wallis tests were applied to test for any differences in the distribution of ages between more than two subgroups of patients defined by histology. To assess marginal homogeneity as well as concordance of serum marker elevation rates, McNemar's test and Cohen's kappa statistics were employed. A kappa value below 0.60 indicates a significant level of disagreement. P-values less than 0.05 were considered as statistically significant in this paper. Data on serum tumor marker expressions particularly for M371 were missing in about one third of patients. Thus, the statistical analyses employed varying sample sizes according to the available entries.

## Results

### General results

**Fig. 1:**
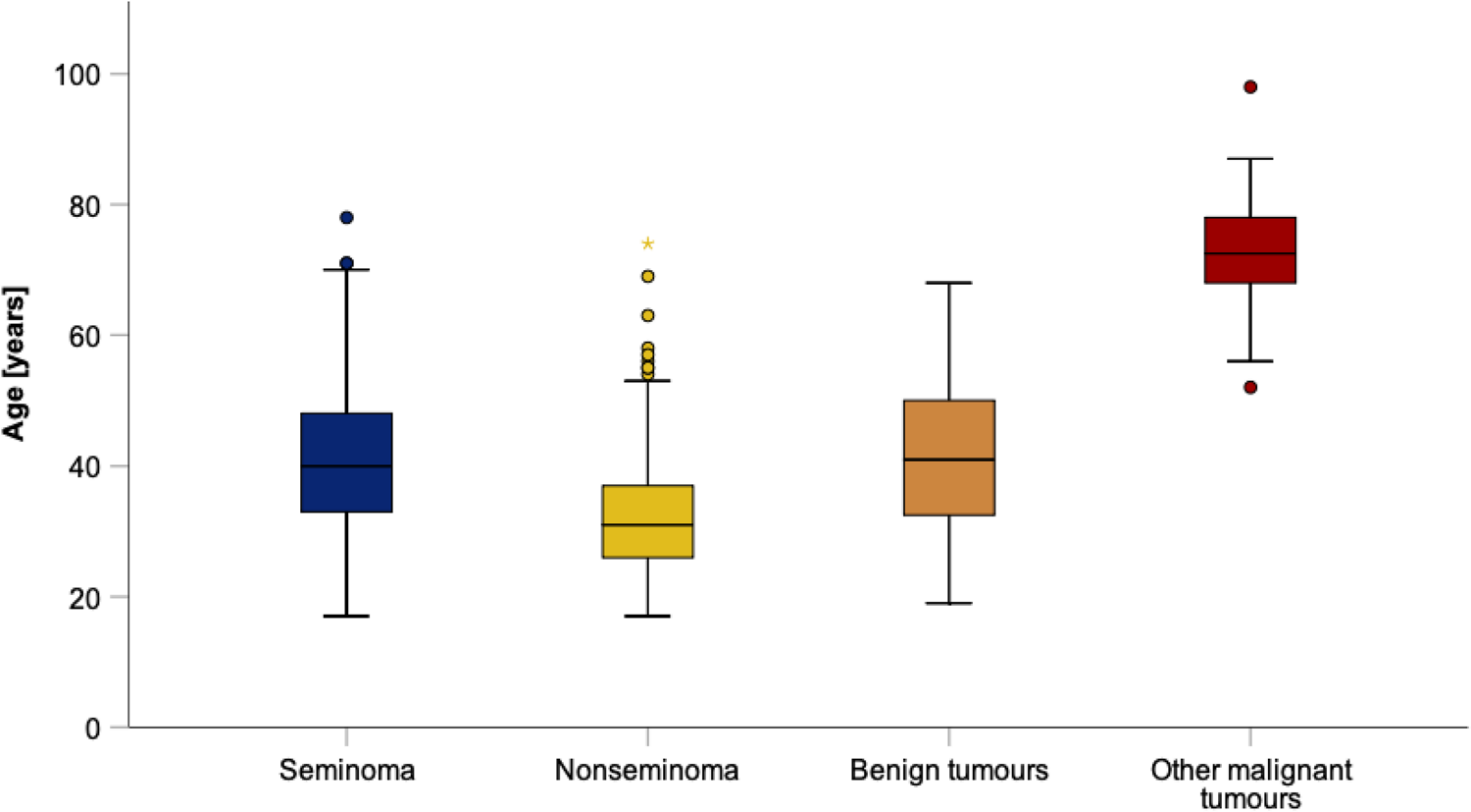
Overview of age distributions in four histologic subgroups of testicular neoplasms. Box–Whisker plots showing the distribution of patient ages stratified by histologic subtypes of testicular neoplasms. The box displays the first quartile, median and third quartile. The whiskers are defined as the largest or lowest observed value that falls within the 1.5 times the interquartile range measured from Q3 or Q1, respectively. Dots represent outliers

A total of 641 patients with a median age of 38 years were enrolled. The frequencies of the four histologic subgroups with corresponding median ages are outlined in Table 1 and Fig. 1. Part of this descriptive analysis had been reported earlier (Dieckmann et al. 2022a). The distribution of ages is significantly different by overall comparison across histologic groups (*p* < 0.001, Kruskal–Wallis test). Benign tumors (BT) comprised of gonadal stromal tumors for the most part and benign epidermoid cysts and other rare neoplasms to a lesser degree. Other malignant tumors (OM) comprised of various forms of malignant testicular lymphoma. Age, histology, and clinical staging were available in all patients. Marker elevations regarding AFP and bHCG were available in 640 patients, regarding LDH in 633 cases, and with respect to M371 in 451 patients.

**Table 1:** Patient population, frequencies of histologic subgroups and corresponding ages

	*n* (% of all)	min	Age (years)	Med	Q3	max
			Q1			
Total population	641 (100%)	17	31	38	47	98
Seminoma	365 (56.94%)	17	33	40	48	78
Nonseminoma	179 (27.93%)	17	26	31	37	74
Benign tumours	79 (12.32%)	19	32	41	50	68
Other malignant tumours	18 (2.81%)	52	68	72.5	78	98

Kruskal–Wallis Test *p* < 0.001 (for overall comparison of groups regarding age)

### Association of patient age with histology

The frequencies of the histologic subgroups stratified in four categories of patient age are listed in Table 2. Overall, the frequencies of histologic subgroups are significantly different among the age categories (*p* < 0.0001, chi-square test). In the youngest age category, nonseminoma represents the most frequent subtype with 61.5%. In the age category 41–50 years, seminoma has the highest proportion with 73.1%. All patients of the OM subgroup are aged > 50 years. Table 3 lists the results of statistical comparisons of particular subgroups regarding the frequencies of age categories. Nonseminoma is more prevalent in the younger age categories than seminoma (*p* < 0.0001), while germ cell tumors as a whole (SE + NS) are not statistically different from benign tumors regarding the distribution of age categories.

**Table 2:** Frequencies of histologic subgroups in various age categories

Age categories (years)	*n*	SE (%)	NS (%)	BT (%)	OM (%)
≤ 30	143 (100%)	40 (27.97%)	88 (61.54%)	15 (10.49%)	0 (0.00%)
31–40	229 (100%)	147 (64.19%	58 (25.33%)	24 (10.48%)	0 (0.00%)
41–50	156 (100%)	114 (73.08%)	21 (13.46%)	21 (13.46%)	0 (0.00%)
> 50	113 (100%)	64 (56.64%)	12 (10.62%)	19 (16.81%)	18 (15.93%)

Chi-Square Test *p* < 0.0001 (for overall distribution of histologic subgroups among age categories)

*SE* seminoma, *NS* nonseminoma, *BT* benign tumors, *OM* other malignant tumors

**Table 3:** Comparisons of histologic subgroups with regard to distribution of age categories (*p* values) according to data listed

Histologic group	Compared with (histologic group)	*P* value*
Seminoma (SE)	Nonseminoma (NS)	< 0.0001
GCT (SE + NS)	Non-GCT (BT + OM)	< 0.0001
GCT (SE + NS)	Benign tumors (BT)	0.0977
GCT (SE + NS)	Other malignant tumors (OM)	< .0001

*Chi-Square Test, *GCT* germ cell tumor

### Serum tumor marker expressions vary among histologic groups

Table 4 outlines the frequencies of elevations of each of the tumor markers (expression rates) in the four histologic subgroups. The highest observed rate represents 93.6% for M371 in nonseminoma. The expression rates of each of the markers are statistically different among the histologic subgroups by overall comparison. The results of statistical comparisons of marker expression rates among particular histologic subgroups are listed in (Table 5). The difference between SE and NS regarding M371 expression is statistically significant (82.7% versus 93.6%, chi-square test, *p* = 0.0061).

**Table 4:** Tumor marker expression rates in histologic subgroups

	bHCG	AFP	LDH	AFP/ bHCG	M371
	n/N (%) [95% CI]	n/N (%) [95% CI]	n/N (%) [95% CI]	n/N (%) [95% CI]	n/N (%) [95% CI]
SE (*n = *365)	106/364 (29.12%) [24.50%, 34.08%]	19/364 (5.22%) [3.17%, 8.03%]	82/362 (22.65%) [18.44%, 27.32%]	122/364 (33.52%) [28.68%, 38.62%]	215/260 (82.69%) [77.54%, 87.09%]
NS (*n = *179)	100/179 (55.87%) [48.27%, 63.27%]	97/179 (54.19%) [46.59%, 61.64%]	40/174 (22.99%) [16.96%, 29.96]	123/179 (68.72%) [61.37%, 75.42%]	102/109 (93.58%) [87.22%, 97.38%]
BT (*n = *79)	2/79 (2.53%) [0.31%, 8.85%]	2/79 (2.53%) [0.31%, 8.85%]	1/79 (1.27%) [0.03%, 6.85%]	4/79 (5.06%) [1.40%, 12.46%]	5/71 (7.04%) [2.33%, 15.67%]
OM (*n = *18)	0/18 (0.00%) [0.00%, 18.53%]	0/18 (0.00%) [0.00%, 18.53%]	2/18 (11.11%) [1.38%, 34.71%]	0/18 (0.00%) [0.00%, 18.53%]	3/10 (30.00%) [6.67%, 65.25%]

CI represent exact Clopper–Pearson confidence interval; N number of patients eligible

AFP/bHCG elevation of either AFP or bHCG or of both markers

**Table 5:** Comparisons of tumor maker expression rates among histologic subgroups (*p* values) according to data listed

	bHCG *p*	AFP *p*	LDH *p*	AFP/ bHCG *p*	M371 *p*
overall	< 0.0001	< 0.0001	0.0001	< 0.0001	< 0.0001
SE vs. NS	< 0.0001	< 0.0001	0.9307	< 0.0001	0.0061
SE + NS vs. (OM + BT)	< 0.0001	< 0.0001	< 0.0001	< 0.0001	< 0.0001

*p* values relate to chi-Square Test for comparison of frequencies of marker elevation rates across histologic subgroups; AFP/bHCG elevation of either AFP or bHCG or of both markers

In nonseminoma, the expression rates of AFP/bHCG and M371 are not much different from each other with 68.7% and 93.6%, respectively. To test for agreement of NS cases expressing M371 and AFP/ bHCG at the same time, we employed McNemar's test by analyzing 109 patients where both markers were available (Table 6). In this setting, the expression rates of AFP/bHCG and M371 were 69.72% and 93.58%, respectively. Testing for marginal homogeneity revealed a significant difference between the two rates (*p* < 0.0001, McNemar). The low Cohen's kappa coefficient of 0.11 further demonstrates the large disagreement between the two markers. Also, the 95% CIs of the frequencies of AFP/bHCG (61.37–75.42%) and M371 (87.22–97.38%) do not overlap. Thus, the M371 expression rate in nonseminoma is clearly superior to the combined measurement of classical markers (AFP/bHCG).

**Table 6:** Detailed comparison of expression rates of AFP/bHCG and M371 in nonseminoma by analyzing the cases where both values are available (*n = *109)

*n* (%)	M371		Overall AFP/bHCG expression
AFP/bHCG	Negative	Positive	
Negative	4 (3.67%)	29 (26.61%)	33 /109 (30.28%)
Positive	3 (2.75%)	73 (66.97%)	76 /109 (69.72%)
Overall M371 express/bHCGM371ion	7 /109 (6.42%)	102/109 (93.58%)	109 (109 (100%)
*p* value*	< 0.0001		
Agreement, *n* (%)	77 /70.64%)		
Disagreement, *n* (%)	32 (29.36%)		
κ**, 95% CI	0.1052 [-0.05; 0.26]		

*McNemar’s test assessed the similarity of the overall (marginal) rates of marker positivity/negativity between the two markers

**Cohen’s kappa assessed the agreement between/concordance of the expression of the two markers

Benign tumors and other malignant tumors revealed elevations of tumor markers only in isolated cases. But of note, a 30% expression rate of M371 was found in other malignant tumors; however, the confidence intervals are extremely wide (6.67%—65.25%).

### Association of tumor marker expression rates with clinical staging in germ cell tumors

The expression rates of the serum tumor markers in the four clinical stages of GCTs are listed in Table 7. There is a clear and statistically significant increase of expression rates with increasing clinical stages with respect to each of the markers (all p < 0.001; Cochran–Armitage trend test). Noteworthy, in all metastasized stages (> CS1), M371 revealed a 100% expression rate (95% CIs 94.22 – 100%) while the rate of AFP/bHCG is 66.7% (95% CIs 58.27–74.94%). In all clinical stages, the expression rate of M371 is clearly superior to that of each of the classical markers and also to combined measurement of markers.

**Table 7:** Frequencies of tumor marker expression rates in clinical stages of testicular germ cell tumors (SE + NS)

	bHCG	AFP	LDH	AFP/ bHCG	M371
	n/N (%) [95% CI]	n/N (%) [95% CI]	n/N (%) [95% CI]	n/N (%) [95% CI]	n/N (%) [95% CI]
CS1 (*n = *421)	136/420 (32.38%) [27.92%, 37.09%]	71/420 (16.90%) [13.45%, 20.84%]	66/417 (15.83%) [12.46%, 19.69%]	163/420 (38.81%) [34.12%, 43.65%]	255/307 (83.06%) [78.39%, 87.08%]
CS2a/2b (*n = *84)	43/84 (51.19%) [40.04%, 62.26%)	28/84 (33.33%) [23.42%, 44.46%]	27/80 (33.75%) [23.55%, 45.19%]	51/84 (60.71%) [49.45%,71.20%)	51/51 (100%) [93.02%, 100%]
CS2c (*n = *15)	10/15 (66.67%) [38.38%,88.18%]	3/15 (20.00%) [4.33%, 48.09%]	10/15 (66.67%) [38.38%,88.18%]	12/15 (80.00%) [51.91%, 95.67%]	3/3 (100%) [29.24%, 100%]
CS3 (*n = *24)	17/24 (70.83%) [48.91%, 87.38%]	14/24 (58.33%) [36.64%, 77.89%]	19/24 (79.17%) [57.85%, 92.87%]	19/24 (79.17%) [57.85%, 92.87%]	8/8 (100%) [68.77%, 100%]
*p* value*	< 0.0001	< 0.0001	< 0.0001	< 0.0001	0.0009

CI represent exact Clopper–Pearson confidence interval; AFP/bHCG elevation of either AFP or bHCG or of both markers; *Cochran–Armitage Trend Test; N number of patients eligible; p values relate to comparisons of marker frequencies among clinical stages

### Association of age with serum marker expression rates

The expression rates of each of the tumor markers stratified by four age categories found in the entire study population (all histologic groups) are listed in Table 8. The expression rates of bHCG, AFP, and M371 are significantly different among age categories, but this not true for LDH (chi-square test). The trend of increasing expression rates in decreasing (younger) age is confirmed for bHCG, AFP, and M371, but not for LDH (Cochran–Armitage trend test). If only GCT patients are considered (Table 9), significantly different expression rates among age categories are only found for bHCG and AFP but not for LDH and M371 (chi-square test). However, the trend towards higher expression rates with decreasing age is found for bHCG, AFP, and also M371. This statistical trend is only weakly significant in M371 (*p* = 0.04), probably because all age categories revealed high expression rates of M371 above 80%, yet with a slight increase to 92.6% in the youngest age category.

**Table 8:** Frequencies of tumor marker expression rates in four age categories of all patients with testicular neoplasms (SE + NS + BT + OM)

	bHCG	AFP	LDH	AFP/bHCG	M371
Age (years)	n/N (%) [95% CI]	n/N (%) [95% CI]	n/N (%) [95% CI]	n/N (%) [95% CI]	n/N (%) [95% CI]
≤ 30 (*n = *143)	68/143 (47.55%) [39.15%, 56.06%]	52/143 (36.36%) [28.49%, 44.25%]	34/142 (23.94%) [17.19%, 31.82%]	77/143 (53.85%) [45.68%, 62.02%]	75/96 (78.13%) [68.53%, 85.92%]
31–40 (*n = *229)	67/229 (29.26%) [23.45%, 35.61%]	39/229 (17.03%) [12.40%, 22.54%]	35/226 (15.49%) [11.03%, 20.87%]	83/229 (36.24%) [30.02%, 42.47%]	128/166 (77.11%) [69.96%, 83.26%]
41–50 (*n = *156)	46/155 (29.68%) [22.62%, 37.53%]	15/155 (9.68%) [5.52%, 15.46%]	36/154 (23.38%) [16.94%, 30.86%]	54/155 (34.84%) [27.37%, 42.90%]	76/109 (69.72%) [60.19%, 78.16%]
> 50 (*n = *113)	27/113 (23.89%) [16.37%, 32.83%]	12/113 (10.62%) [5.61%, 17.82%]	20/111 (18.02%) [11.37%, 26.45%]	35/113 (30.97%) [22.61%, 40.36%]	46/79 (58.23%) [46.59%, 69.23%]
*p* value*	0.0002	< .0001	0.1296	0.0004	0.0085
*p* value**	0.0002	< .0001	0.6792	0.0001	0.0014

CI represent exact Clopper–Pearson confidence intervals; AFP/bHCG elevation of either AFP or bHCG or of both markers; *Chi-Square Test (relates to overall distribution of marker frequencies among age categories); **Cochran–Armitage Trend test (test for trend across age categories); N number of patients eligible

**Table 9:** Frequencies of tumor marker expression rates in four age categories of patients with testicular germ cell tumors (SE + NS)

	bHCG	AFP	LDH	AFP/ bHCG	M371
Age (years)	n/N (%) [95% CI]	n/N (%) [95% CI]	n/N (%) [95% CI]	n/N (%) [95% CI]	n/N (%) [95% CI]
≤ 30 (*n = *128)	68/128 (53.13%) [44.11%, 62.00%]	52/128 (40.63%) [32.04%, 49.66%]	34/127 (26.77%) [19.31%, 35.35%]	77/128 (60.16%) [51.13%, 68.70%]	75/81 (92.59%) [84.57%, 97.23%]
31 – 40 (*n = *205)	67/205 (32.68%) [26.31%, 39.56%]	39/205 (19.02%) [13.89%, 25.08%]	34/202 (16.83%) [11.95%, 22.72%]	83/205 (40.49%) [33.71%, 47.55%]	126/146 (86.30%) [89.64%, 91.43%]
41 – 50 (*n = *135)	44/134 (32.84%) [24.97%, 41.47%]	14/134 (10.45%) [5.83%, 16.91%]	36/133 (27.07%) [19.73%, 35.45%]	51/134 (38.06%) [29.82%, 46.84%]	74/91 (81.32%) [71.78%, 88.72%]
> 50 (*n = *76)	27/76 (35.53%) [24.88%, 47.34%]	11/76 (14.47%) [7.45%, 24.42%]	18/74 (24.32%) [15.10%, 35.69%]	34/76 (44.74%) [33.31%, 56.5 9%]	42/51 (82.35%) [ 6 9.13%, 91.60%]
*P* value*	0.0008	< .0001	0.0818	0.0010	0.1629
*P* value**	0.0068	< .0001	0.7560	0.0098	0.0400

CI represent exact Clopper–Pearson confidence intervals; AFP/bHCG elevation of either AFP or bHCG or of both markers; *Chi-Square Test; **Cochran–Armitage Trend test; N number of patients eligible

## Discussion

The present study provides clear evidence for significant associations of serum tumor marker expression rates with histology, patient age, and with clinical staging in patients with testicular neoplasms. In GCTs, marker expression rates increase with clinical stages. The novel marker M371 significantly outperforms the classical serum markers AFP, bHCG and LDH. Also, younger patient age is significantly associated with higher serum marker expression rates. The present study confirmed current clinical experience but also expanded and corroborated existing knowledge by analyzing a large sample of contemporary and unselected patients and by employing comprehensive statistical analysis, thus providing a high level of formal evidence.

### Association of age with histology of testicular tumor

The median ages of seminoma (SE), nonseminoma (NS), benign tumors (BT) and other malignant tumors (OM) are significantly different from each other. Conversely, the proportions of the four histologic subgroups vary significantly among age categories. Of note, malignancies other than germ cell tumors (OM) were only observed in patients aged > 50 years. This observation relates to malignant lymphoma, which represents the only histologic entity found in the OM group and this malignancy typically presents in patients aged > 60 years (Xu and Yao 2019; Koch et al. 2022). In the youngest age category (≤ 30 years), nonseminoma revealed the highest prevalence with 61.5%, while in the oldest age category (> 50 years), seminoma ranked first comprising of 56.6%. Benign tumors (BT) showed almost equal frequencies in all four age categories. These results are in accordance with the classical data reported from the large population of the British Testicular Tumour Panel (*n = *1812) where mean ages of 41.2 years; 29.8; 59.8; and 33.3—47.5 years were noted in seminomas, nonseminomas, malignant lymphoma, and benign gonadal stromal tumors, respectively (Pugh 1976). The significantly younger median age of nonseminoma in comparison to seminoma is settled knowledge since decades and reflects the biologic difference between the two entities (Friedman and Moore 1946). The BT group of the present study consisted of Leydig cell tumors for the major part, and the median age of 41 years found in this group is identical with the median age of 41 years reported in a recent study on 208 cases with Leydig cell tumors (Ruf et al. 2020). However, this study also pointed out that Leydig cell tumors may occur at any age between 17 and 81 years and this observation is in line with the age distribution found in the present investigation. Likewise, a study from Wessex, UK, reported an arithmetical mean age of benign gonadal stromal tumors of 43 years but in view of the wide range of 18 – 79 years, no particular age predisposition was noted (Featherstone et al. 2009). An Italian study on small testicular tumors occurring in 64 infertile males reported a median age of 40 years in patients with benign tumors compared to the significantly younger age of 36 years in malignant tumors (Gobbo et al. 2022). Similarly, an Austrian study reported mean ages of 41.1 years and 32.5 years in benign and malignant tumors, respectively (Staudacher et al. 2020). However, the latter two studies probably involve selection bias. The Italian study had selectively enrolled patients with infertility which is rarely encountered in patients aged > 50 years. The Austrian study had solely included patients with tumors sized < 2 cm. By contrast, the present patient population comprises of unselected patients of all ages and of testicular new growths of all sizes. In all, patient age is significantly associated with histology of testicular neoplasms and this result suggests that age may be involved in pathogenetic processes of testicular neoplasms (Stang et al. 2023). With respect to diagnostic work-up of testicular masses, age is yet of limited value, since GCTs and benign tumors may occur at any age.

### Association of tumor marker expression rates with histology

There are significant differences of expression rates of the markers bHCG, AFP, LDH, and M371 among the four histologic groups. The expression rate of bHCG in seminoma of 29.1% observed in this study is not much different from the rate of 35% observed in a pivotal series from 1997 (Weissbach et al. 1997) and also consistent with the rates of 18.8%–31.8% reported in a contemporary review (Dieckmann et al. 2019b). Noteworthy, AFP was found to be elevated in 5.2% of seminoma patients. Although AFP production in pure seminoma is biologically not possible, clinical series repeatedly reported slightly elevated AFP levels in pure seminoma with no changes despite curative treatment. The present results are in accordance the international consensus that mildly elevated AFP levels with no clinical significance may occur in isolated cases with seminoma (Dieckmann et al. 2017; Wymer et al. 2017; Brandt et al. 2022). In nonseminoma, bHCG and AFP expression showed rates of 55.8% and 54.2%, respectively. These results are consistent with rates of 52.9%–63.6% and 55.1%–70% for bHCG and AFP, respectively, reported in contemporary clinical series (Germa-Lluch et al. 2002; Dieckmann et al. 2019b; Neumann et al. 2011).

The novel marker M371 outperforms all classical markers with expression rates of 82.7% and 93.6% in seminoma and nonseminoma, respectively, and these rates are also significantly higher than the combined rate of AFP and/ or bHCG in nonseminoma (68.7%).

This result is in line with all previous reports on M371 (Dieckmann et al. 2019a; Mørup et al. 2020; Sequeira et al. 2022; Syring et al. 2015; Murray et al. 2018; Piao et al. 2021) and it clearly underscores the usefulness of the M371 test reiterating the demand for its prompt clinical implementation (Leão et al. 2021). Noteworthy, we found a modest but significantly lower expression rate of M371 in seminoma compared to nonseminoma (82.7% versus 93.6%) which had been documented in most of the recent reports (van Agthoven and Looijenga 2017; Dieckmann et al. 2019a; Mørup et al. 2020) but not in all of them (Myklebust et al. 2021; Vilela-Salgueiro et al. 2018; Mego et al. 2019).

Expectedly, no significant elevations of serum markers were observed in benign tumors, since AFP, bHCG and M371 represent specific products of embryonic tissues that are only present in GCTs. Yet, we observed marker elevations in isolated patients with benign tumors, but there is no information about the extent of level elevations. This result is consistent with a previous series (Belge et al. 2020). Most probably, these elevations represent idiopathic unspecific elevations similar to the AFP elevations in seminoma (Dieckmann et al. 2017; Wymer et al. 2017). Of note is the elevation of M371 levels in 30% of other malignancies. Such elevations had been found previously in some cases with testicular malignant lymphoma (Belge et al. 2020; van Agthoven and Looijenga 2017). Most of the lymphoma cases with M371 elevations had extended disease. In the present study, only three of ten lymphoma cases had such elevations, and in light of the wide confidence limits (6.6%–65.3%), chance effects must be considered. However, the repeated detection of elevated M371 levels in malignant lymphoma may raise the hypothesis that M371 elevations may result not only from GCTs but possibly also from other neoplasms or from other diseases such as Covid 19 infection as recently reported (Goebel et al. 2022). In a previous study, isolated cases with non-testicular malignancies were shown to have M371 levels in the range of RQ 5–12, thus slightly above the ULN of RQ = 5. As some of the healthy controls also had levels of that extent, those elevations were not considered to represent a true-positive result for M371 (Spiekermann et al. 2015). In the present study, the value of RQ = 5 was considered as ULN by default. As shown in a recent study on residual masses subsequent to chemotherapy in seminoma, some patients without active disease had values slightly above the RQ = 5 threshold (Dieckmann et al. 2022b). Thus, it is currently unclear, if the ULN of 5 of the M371 test is uniformly appropriate. Conceivably, the ULN needs to be set somewhat higher than RQ = 5 and possibly, it needs to be adjusted to the clinical scenario examined. It may be speculated that some of the lymphoma cases of the present study could have had values minimally above the ULN of RQ = 5 and may, thus, represent unspecific marker elevations. Nonetheless, further investigation of M371 expression in malignant lymphoma is required.

### Association of tumor marker expression rates with clinical stages in germ cell tumors

A significant trend towards higher expression rates with increasing clinical stages was shown for each of the four tumor markers. This result mirrors the significant association of marker expression rates with primary tumor-size reported earlier (Dieckmann et al. 2022a, 2019b). The most likely biological explanation for this association is the higher number of marker secreting neoplastic cells in both, increasing primary tumor sizes and increasing clinical stages.

With respect to the classical markers, higher sensitivities in metastasized cases than in localized disease (CS1) had first been documented by Skinner and Scardino in 1981 (Skinner and Scardino 1980) and were confirmed by many others thereafter (Lippert and Javadpour 1981; Bosl et al. 1981; Szymendera et al. 1983; Nørgaard-Pedersen et al. 1984; Fargeot 1990; Daugaard et al. 1990; Kausitz et al. 1992; Weissbach et al. 1997; Trigo et al. 2000; Rothermundt et al. 2018; Dieckmann et al. 2019b). As GCTs as a whole (SE + NS) were evaluated in the present investigation, AFP scored lower expression rates than bHCG which obviously relates to the non-expression of AFP in pure seminoma.

The present data revealed a very high sensitivity of M371 in comparison to the classical markers. In CS1, an elevation of serum levels of AFP and / or bHCG was found in 38.8%, whereas M371 scored more than double with 83.1%. In metastasized cases, M371 is expressed in 100% (95% CIs 94.22–100%) of cases opposed to 66.7% (95% CIs 58.27–74.94%) regarding AFP/bHCG. The confidence intervals do not overlap, and this result is consistent with the significant difference between the expression rates of M371 and AFP/bHCG in nonseminoma documented with McNemar's test. The very high sensitivity of M371 found herein is consistent with previous studies (Dieckmann et al. 2019a; Leão et al. 2021; van Agthoven and Looijenga 2017; Mego et al. 2019; Mørup et al. 2020; Myklebust et al. 2021; Lobo et al. 2019; Nappi et al. 2019; Badia et al. 2021; Ye et al. 2022; Sequeira et al. 2022) underscoring the great superiority of M371 to the classical markers.

### Inverse association of age with serum tumor marker expression rates

There is a significant trend towards higher marker expression rates with younger age for bHCG, AFP, and M371, but not for LDH. This result is found both in the entire population (all histologic groups) and in the GCT subpopulation. The inverse association of marker expression rates with age is probably related to the predominance of AFP- and bHCG-producing nonseminomatous germ cell tumors in the younger age categories. Conversely, seminomas with the majority of which being marker negative, do predominantly occur in older ages (Secondino et al. 2022; Cheng et al. 2018). Thus, the specific predispositions of the various histologic GCT subtypes to different age categories have likely caused the trend towards higher marker expression rates in younger patients. Accordingly, LDH expression is almost equally expressed in all age categories most probably because it is merely related to the number of cell damages and is not specific for any particular histologic GCT subtype.

In GCTs, the trend towards higher expression rate in younger age categories is also seen in M371. This association deserves some consideration, since very high sensitivities of this marker are documented in both seminoma and nonseminoma. However, the sensitivity of M371 is somewhat higher in nonseminoma than in seminoma, and this difference may translate into higher expression rates in younger patients. The inverse age trend is slightly weaker in M371 than in AFP and bHCG as can be noted by the numerically higher p value in M371 (*p* = 0.04) compared to 0.0068 and < 0.0001 regarding bHCG and AFP, respectively. The weaker age trend of M371 does also probably come from the still high M371 expression rate of 82.35% in patients aged > 50 years and the rather small difference to the rate of 92.59% in the youngest age category. The association of marker expression with age had been noted only in one previous report (Dieckmann et al. 2019b). As the association is well explained by the specific age predispositions of the GCT subtypes, that issue had obviously not attracted further investigations.

### Limitations of the study

Selection bias cannot entirely be ruled out in view of the retrospective study design of the present investigation. As the main goal of the study was to examine marker expression rates in relation to clinical factors, the statistical analysis was possibly hampered by lacking marker measurements in a number of patients. With respect to tumor marker elevation rates, only a dichotomized analysis was available in the present study with no further information about the extent of elevations although that information could have endorsed the results. The histological subgroups BT and OM encompassed only small sample sizes which could have limited statistical power despite the overall large patient population. Tumor histologies were based on local pathological examination without expert histopathological review. Probably, a strong point of the study is the large number of 451 measurements of the next-generation tumor marker M371 featuring the usefulness of this diagnostic tool in the clinical management of testicular cancer. Another asset could be the thorough statistical analysis of a large and representative population of patients with testicular neoplasms.

## Conclusions

There are strong interrelations of patient age, histology, and clinical staging with serum tumor marker expression rates in patients with testicular neoplasms. The rates of all markers correlate with tumor bulk. The superiority of the new marker M371 to the classical serum markers AFP, bHCG and LDH was confirmed in a large patient sample. Although sporadic elevations of M371 in non-germ cell tumors need further investigation, the present data underscore the exceptional clinical usefulness of the M371 test.

## Data Availability

The datasets analyzed during the current study are available from the corresponding author on reasonable request.

## Abbreviations

AFP: Alpha fetoprotein
AUC: Area under the curve
bHCG: Beta human chorionic gonadotropin
BT: Benign tumors
CI: Confidence interval
CS: Clinical stage
GCT: Germ cell tumor
IQR: Inter-quartile range
LDH: Lactate dehydrogenase
M371: MicroRNA-371a-3p
NS: Nonseminoma
n: Number
OM: Other testicular malignancies
SE: Seminoma
Q1: First quartile
Q3: Third quartile
